# Delirium after major trauma critical care and the association with recovery at 12 months

**DOI:** 10.1177/14604086251343660

**Published:** 2025-06-19

**Authors:** Robert Christie, Elaine Cole

**Affiliations:** 1 Royal London Hospital, London, UK; 2 Blizard Institute, 4617 Queen Mary University of London, London, UK

**Keywords:** Delirium, major trauma, critical care, recovery, anxiety, depression, psychological effects

## Abstract

**Purpose:**

Delirium is associated with poor outcomes in general critical care populations, but the effects on recovery in major trauma patients are less clear. This study aimed to characterise critical care trauma patients with self-reported delirium and explore the relationship with recovery at 12 months post-injury.

**Methods:**

A prospective multi-site observational study of patients admitted to four Major Trauma Centre critical care units. Follow-up questionnaires assessed quality of life and recovery using the EQ Visual Analogue Scale (EQ VAS), European Quality-of-Life Five Dimensions (EQ-5D-5L) and the 12-item World Health Organisation Disability Assessment Schedule (WHODAS) 2.0 surveys. Post-discharge support was recorded together with ‘difficulties in the 30 days leading up to follow-up’ (WHODAS).

**Results:**

Of the 285 severely injured adult patients who completed the follow-up questionnaires, 180 (63%) reported delirium in hospital. Traumatic brain injury was higher in the delirium group (40% vs 28%, *p* = 0.05). Overall health score was worse at 12 months for those reporting delirium (EQ-5D VAS: delirium 67.5 vs no delirium 75, *p* = 0.01). Patients with delirium also reported more days where difficulties were present (delirium: 22 days vs no delirium: 17 days, *p* < 0.01) and days of reduced activities (delirium: 7 days vs no delirium: 4 days, *p* = 0.01). Those with in hospital delirium reported more psychological and cognitive problems via both EQ-5D-5L and WHODAS 2.0. Despite this, fewer than half [*n* = 79, (44%)] had received any form of psychological support as part of their treatment or recovery.

**Conclusions:**

Severely injured trauma critical care patients with self-reported in hospital delirium experience worse quality of life at 12 months post-injury. Psychological problems were greater after in-hospital delirium and longer-term support for these patients appears to be limited.

## Introduction

Over a decade ago, a new regional model of major trauma networks was adopted in England and has demonstrated improved survival for severely injured trauma patients.^1,2^ As mortality rates have declined and more patients are surviving previously fatal injuries, there is a growing need to focus on other outcomes, which may adversely affect recovery.^3–6^ Improved survival rates coupled with an ageing population across the developed world mean that severely injured patients are likely to be older than previous trauma populations and experience more complications associated with their primary injury and subsequent care needs.^7^ Many trauma patients who require admission to a critical care unit develop adverse outcomes such as multiple organ dysfunction syndrome (MODS),^8^ infection,^9^ and delirium^10^

Delirium is a syndrome characterised by an acute change or fluctuation in baseline mental status, inattention, and either disorganised thinking or altered level of consciousness.^11^ It is acute in onset and distinct from pre-existing or newly evolving neurological pathology, although the latter may be a contributing and confounding factor. Consensus on diagnostic criteria and recommendations for approved nomenclature of delirium and related terms was agreed within the Statement of Ten Societies.^12^ Polytrauma has been identified as a predisposing risk factor for delirium development,^11^ and this may be worsened in the presence of older age or frailty. The pathophysiology of delirium is uncertain and medical interventions remain largely confined to management of symptoms, with limited effect.^13^ Delirium has been associated with increased mortality,^14–16^ longer critical care and hospital stays^10^ and significantly higher care costs.^17^

The sequelae and longer-term impact of delirium after major trauma are under-reported and remain poorly understood. In critical illness and post-operative recovery cohorts, delirium has been associated with longer-term cognitive impairment beyond hospital discharge.^18–21^ However, the effects on major trauma patients admitted to critical care are unknown. Therefore, the primary aim of this study was to identify the characteristics of adult critical care patients who developed delirium following major traumatic injury. The secondary aim was to explore the relationship between self-reported in-hospital delirium and outcomes at 12 months post-injury.

## Methods

### Study design, sample and setting

This was a multi-site longer-term follow-up analysis of the Multiple Organ Dysfunction in Elderly Trauma (MODET) prospective observational study participants.^8^ Adult trauma patients (age ≥ 16 years) were enrolled into the study on admission to critical care in the four Major Trauma Centres (MTCs – Level 1 equivalent hospitals) within the London Major Trauma System over 18 months from February 2017. Where patient consent was not possible due to incapacity, informed consent was obtained from either a nominated professional consultee and/or a personal consultee until such a time as the patient regained capacity and could be approached for personal consent. Prior to follow-up, the NHS Digital Spine Summary Care Record was checked for each consented patient to ensure no attempts were made to contact those who had died following discharge from hospital. Ethical approval was granted by the Health Research Authority, London, and South East Research Ethics Committee (IRAS 209230).

### Data collection

Follow-up took place between February 2018 and December 2019. At this time, critical care was defined as either an intensive care unit (ICU) offering Level 3 care or a combined unit of intensive (Level 3) care and high dependency (Level 2) care.^22^ Demographic, injury, clinical management and outcome data were collected during the in-hospital phase of the study, and patients were followed up on each day of their critical care admission.^8^ At 12 months post-injury, follow-up questionnaires were either posted or patients were contacted by telephone according to their expressed preference. Over the course of 4 weeks, at least two attempts were made to contact those patients by telephone where this was the expressed preference and initial contact was unsuccessful. Patients were asked about the presence or absence of delirium during their hospital admission. This included any episode of distorted memory or confusion as recollected by the patient or them having been told about such episodes by clinical staff or relatives. Where the patient did not have capacity to respond to the questionnaire, a surrogate completed it on their behalf (almost exclusively next-of-kin). All data were initially collected in paper form via the questionnaire booklet and then converted into an electronic case report form by one of two research nurses using the REDCap (Research Electronic Data Capture) secure, web-based software platform^23,24^ hosted at Queen Mary University of London.

### Outcomes

The primary outcome was patient reported overall health status relating to their recovery at 12 months post-injury. This was measured using the EQ Visual Analogue Scale (VAS), which rates overall health on a 0–100 scale where endpoints are labelled ‘the worst health you can imagine’ through to ‘the best health you can imagine’.^25^ Secondary outcomes included quality of life and functioning at this time point and were measured using two instruments validated for trauma populations. Firstly, the European Quality of Life Five Dimensions (EQ-5D-5L) survey comprises a descriptive system of five dimensions: mobility, self-care, usual activities, pain/discomfort and anxiety/depression with five levels of response ranging from ‘no problems’ to ‘extreme problems’ or ‘unable’.^25^ Secondly, the 12-item World Health Organisation Disability Assessment Schedule (WHODAS) 2.0 survey captures the level of functioning within six domains of life: cognition; mobility; self-care; getting along with other people; life activities; and participation in community activities; with scores of ‘none’ = 0, ‘mild’ = 1, ‘moderate’ = 2, ‘severe’ = 3 and ‘extreme’ = 4.^26^ WHODAS 2.0 also allows the respondent to evaluate the impact of their health condition in terms of duration, where patients are asked how much difficulty they have had in specific areas of functioning during the past 30 days. This is quantified by the number of days that difficulties are present; the number of days when they are totally unable to carry out usual activities due to this; and the number of days when they have had to reduce their usual activities (without ceasing them entirely).^26^ Patients were also asked about any outpatient follow-up and support received from a healthcare professional trained in psychological therapies.

### Data analysis

Data were analysed using Graphpad PRISM (version 9.5.1) and SPSS (version 28). Continuous data were analysed using either unpaired *t* tests or Mann–Whitney *U* tests according to distribution and reported as mean with standard deviation (SD) or medians with interquartile range. Categorical variables were analysed using chi-squared test with Yates’ continuity correction and reported as number and percentage. Patient reported levels of EQ-5D-5L or WHODAS dimensions were compared between delirium and no-delirium groups. A *p*-value of <0.05 was considered significant.

The Strengthening the Reporting of Observational studies in Epidemiology statement checklist^27^ was used to report the results.

## Results

Of the 1316 patients enrolled into MODET, 707 patients consented to be followed-up at 12 months post-injury, and of these, 285 (40%) completed the delirium questions and outcome instruments. There were 71 surrogates who completed the questionnaire on behalf of the patient, of which 62 (88%) were a partner or direct family member. Three quarters of patients were male, and one-third were aged 65 years or more (34%) (Table 1). The majority had suffered blunt trauma (93%), of which the most common mechanism of injury was road traffic collision (43%). The cohort were severely injured [median Injury Severity Score (ISS) 25 (IQR 16–43)], and just over a third of respondents (36%) had sustained a traumatic brain injury (TBI). Infection developed in a quarter of patients (23%), two-thirds (61%) experienced MODS, and the median critical care stay was 9 days (IQR 4–17.5).

**Table 1.** Patient characteristics and outcomes.

	All (*n* = 285)	Delirium (*n* = 180)	No delirium (*n* = 105)	*p*-value
Male sex Female sex	213 (75) 72 (25)	137 (76) 43 (24)	76 (72) 29 (28)	0.48
Age, years	55 (36–71)	55 (32–66)	54 (38–72)	0.26
16–44 years 45–64 years 65–74 years ≥75 years	101 (35) 88 (31) 49 (17) 47 (16)	66 (37) 55 (31) 31 (17) 28 (16)	35 (33) 33 (31) 18 (17) 19 (18)	0.96
Pre-injury frailty	39 (14)	25 (14)	14 (13)	0.99
Comorbidities	3 (0–5)	4 (0–5)	2 (0–4)	0.21
Blunt trauma	266 (93)	170 (94)	96 (91)	0.33
Road traffic collision Fall ≥ 2 m Fall < 2 m Other	122 (43) 69 (24) 39 (14) 55 (19)	79 (44) 42 (23) 24 (13) 35 (19)	43 (41) 27 (26) 15 (14) 20 (19)	0.95
Traumatic brain injury	102 (36)	72 (40)	30 (28)	0.05
APACHE II score	12 (9–17)	13 (9–17)	11 (8–17)	0.06
Injury severity score	25 (16–34)	25 (17–33)	25 (13–36)	0.99
Infection	66 (23)	43 (24)	23 (22)	0.77
MODS	175 (61)	120 (67)	55 (52)	0.02
Ventilator days	7 (6–8)	7 (6–9)	7 (5–8)	0.81
Critical care stay (days)	9 (4–17.5)	12 (5–19.75)	7 (4–12.5)	<0.01
Hospital stay (days)	28 (16–43)	30 (18–46)	22 (12.5–38)	<0.01
Post discharge outpatient follow-up	242 (86)	160 (90)	82 (79)	0.01
Post discharge trained psychological support	117 (41)	79 (44)	38 (36)	0.21

Data are presented as *n* (%) or median (IQR). *p*-value signifies statistical comparisons between no delirium and delirium groups (Mann–Whitney *U* tests or chi-square tests).

APACHE II: Acute Physiology and Chronic Health Evaluation score; ISS: Injury Severity Score; MODS: multiple organ dysfunction syndrome; LoS: length of stay.

Overall, two-thirds (63%) of respondents reported having distorted memories during hospitalisation or had been told that they were delirious at some point whilst in hospital. Nearly all of patients in the delirium group reported some degree of amnesia whilst in critical care compared to three quarters of the no-delirium patients (delirium: 94% vs no delirium: 73%, *p* < 0.01). Explicit recollections of the symptoms of delirium (e.g., hallucinations, vivid dreams, nightmares and misperceptions) were rarely mentioned by patients, and where they were, it was often as a vague memory of something ‘troublesome’, which lingered beyond hospital discharge and, for a small minority, was still troubling at follow-up.

There were few differences in demographics and injury characteristics between the two groups other than a higher rate of TBI in the delirium group (delirium: 40% vs no delirium: 28%, *p* = 0.05). Outcomes differed where those with delirium had a higher incidence of MODS (delirium: 67% vs no delirium 52%, *p* = 0.02), and longer critical care (delirium: 12 days vs no delirium: 7 days, *p* < 0.01) and hospital stays (delirium: 30 days vs no delirium: 22 days, *p* = 0.001).

Most patients had received injury-related outpatient follow-up following hospital discharge, but this was higher in the delirium group (delirium: 90% vs no delirium: 79%, *p* = 0.01). However, less than half of patients in either group received any support from professionals trained in psychological therapies (e.g., a counsellor, psychologist, or psychiatrist) (delirium: 44% vs no delirium: 36%, *p* = 0.21; Table 1).

**Figure 1.**
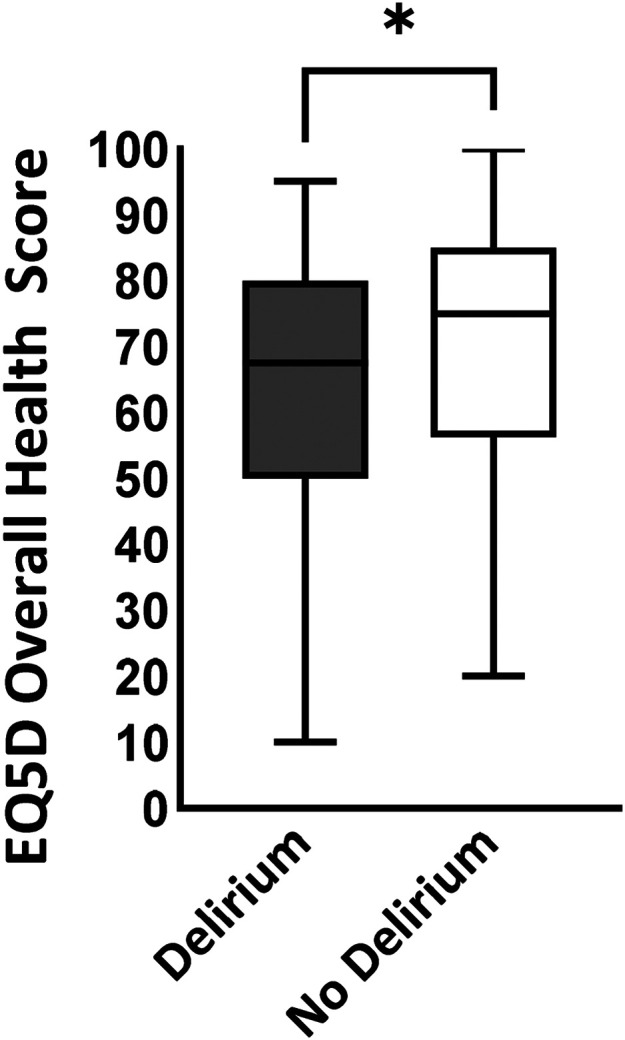
EQ-5D-5L overall health status score at 12 months post injury (visual analogue score out of 100; median, IQR) for delirium and no delirium groups. *p = 0.01.

At 12 months post-injury, overall health status was worse for patients in the delirium group (delirium: EQ-5D VAS 67.5 vs no delirium: EQ-5D VAS 75, *p* = 0.01; Figure 1).

**Figure 2.**
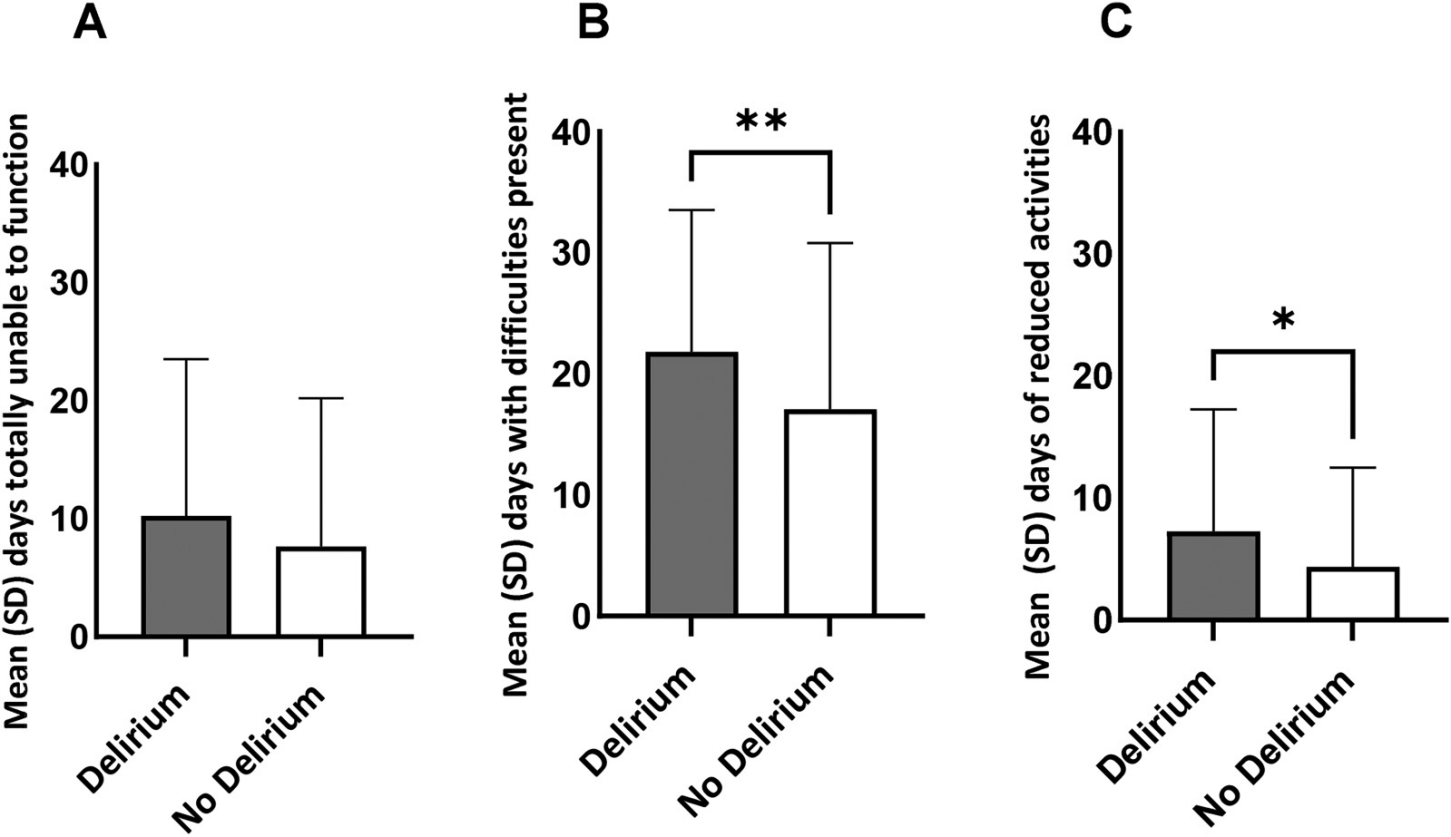
WHODAS 2.0 functioning during the past 30 days for delirium and no delirium groups. (A) Days of total inability to complete activities. (B) Days with difficulty in activities present. (C) Days of reduced or cut back activities. **p = 0.002; *p = 0.01.

Patients being unable to function at all within the 30-day period leading up to follow-up did not differ between groups (Figure 2A). However, the number of days where difficulties were present was greater for the delirium group (delirium: 22 days vs no delirium: 17 days, *p* < 0.01; Figure 2B), and there were almost twice as many days where activities had to be reduced at 12 months post-injury (delirium: 7 days vs no delirium: 4 days, *p* = 0.01; Figure 2C).

Analysis of the EQ-5D-5L and WHODAS 2.0 domains did not identify physical factors that may have contributed to the reported reduced quality of life at this point in recovery. In the EQ-5D-5L results, there were few differences between groups except for anxiety and depression, which was worse with those who had experienced delirium at all levels except severe (Table 2; *p* = 0.04). There was also a trend towards more moderate and severe emotional and concentration problems at 12 months according to WHODAS 2.0, but no differences in other domain functions (Table 3).

**Table 2.** Self-reported functional outcomes using EQ-5D-5L.

**EQ-5D-5L domain**	**Delirium (*n*** **=** **180)**	**No delirium (*n*** **=** **105)**	***p*-value**
**Mobility** I have **no** problems in walking about I have **slight** problems in walking about I have **moderate** problems in walking about I have **severe** problems in walking about I am **unable** to walk about	53 (29) 40 (22) 47 (26) 27 (15) 13 (7)	42 (40) 20 (19) 24 (23) 8 (8) 11 (10)	0.15
**Self-care** I have **no** problems washing or dressing I have **slight** problems washing or dressing I have **moderate** problems washing or dressing I have **severe** problems washing or dressing I am **unable** to wash or dress	91 (51) 36 (20) 27 (15) 14 (8) 12 (7)	66 (63) 14 (13) 13 (12) 5 (5) 7 (7)	0.31
**Usual activities** I have **no** problems doing my usual activities I have **slight** problems doing my usual activities I have **moderate** problems doing my usual activities I have **severe** problems doing my usual activities I am **unable** to do my usual activities	37 (21) 39 (22) 42 (23) 37 (21) 25 (14)	36 (34) 18 (17) 24 (23) 13 (12) 14 (13)	0.08
**Pain/discomfort** I have **no** pain or discomfort I have **slight** pain or discomfort I have **moderate** pain or discomfort I have **severe** pain or discomfort I have **extreme** pain or discomfort	45 (25) 56 (31) 45 (25) 29 (16) 5 (3)	33 (31) 31 (30) 23 (22) 16 (15) 2 (2)	0.81
**Anxiety/depression** I am **not** anxious or depressed I am **slightly** anxious or depressed I am **moderately** anxious or depressed I am **severely** anxious or depression I am e**xtremely** anxious or depressed	58 (33) 41 (23) 54 (30) 15 (8) 10 (6)	49 (47) 21 (20) 22 (21) 11 (11) 1 (1)	0.04

Data are displayed as *n* (%). Data compared with chi-square tests. Missing responses in Anxiety/depression domain: *Delirium* = 2, *No Delirium* = 1.

**Table 3.** Self-reported functional outcomes using World Health Organisation Disability Assessment Schedule (WHODAS) 2.0.

**WHODAS functional outcome**	**Delirium (*n*** **=** **180)**	**No delirium (*n*** **=** **105)**	***p*-value**
**S1. Difficulty standing for long periods such as 30 min** No difficulty Mild difficulty Moderate difficulty Severe difficulty Extreme difficulty/cannot do	60 (33) 32 (18) 27 (15) 20 (11) 41 (23)	44 (42) 11 (10) 19 (18) 12 (11) 19 (18)	0.31
**S2. Difficulty taking care of household responsibilities** No difficulty Mild difficulty Moderate difficulty Severe difficulty Extreme difficulty/cannot do	62 (35) 31 (17) 35 (20) 28 (16) 23 (13)	47 (45) 15 (14) 24 (23) 6 (6) 13 (12)	0.09
**S3. Difficulty learning a new task** No difficulty Mild difficulty Moderate difficulty Severe difficulty Extreme difficulty/cannot do	85 (47) 34 (19) 29 (16) 20 (11) 12 (7)	60 (58) 14 (13) 13 (13) 11 (11) 6 (6)	0.51
**S4. Difficulty joining in community activities** No difficulty Mild difficulty Moderate difficulty Severe difficulty Extreme difficulty/cannot do	63 (36) 31 (18) 37 (21) 26 (15) 20 (11)	53 (50) 16 (15) 13 (12) 12 (11) 11 (10)	0.13
**S5. How much have you been emotionally affected by your health problem?** No difficulty Mild difficulty Moderate difficulty Severe difficulty Extreme difficulty/cannot do	27 (15) 36 (20) 54 (30) 54 (30) 8 (5)	29 (28) 25 (24) 25 (24) 21 (20) 5 (5)	0.05
**S6. Difficulty concentrating on doing something for ten minutes** No difficulty Mild difficulty Moderate difficulty Severe difficulty Extreme difficulty/cannot do	77 (43) 38 (21) 35 (19) 23 (13) 7 (4)	61 (58) 16 (15) 12 (11) 9 (9) 7 (7)	0.05
**S7. Difficulty walking a long distance** No difficulty Mild difficulty Moderate difficulty Severe difficulty Extreme difficulty/cannot do	65 (36) 23 (13) 23 (13) 18 (10) 51 (28)	41 (39) 14 (13) 12 (11) 17 (16) 21 (20)	0.38
**S9. Difficulty getting dressed** No difficulty Mild difficulty Moderate difficulty Severe difficulty Extreme difficulty/cannot do	91 (51) 33 (18) 30 (17) 11 (6) 15 (8)	66 (63) 13 (12) 14 (13) 3 (3) 9 (9)	0.26
**S10. Difficulty dealing with people you do not know** No difficulty Mild difficulty Moderate difficulty Severe difficulty Extreme difficulty/cannot do	90 (50) 36 (20) 29 (16) 19 (11) 5 (3)	64 (62) 13 (13) 16 (16) 7 (7) 3 (3)	0.30
**S11. Difficulty maintaining a friendship** No difficulty Mild difficulty Moderate difficulty Severe difficulty Extreme difficulty/cannot do	102 (57) 30 (17) 24 (13) 17 (10) 5 (3)	67 (64) 11 (10) 14 (13) 8 (8) 5 (5)	0.50
**S12. Difficulty doing your day-to-day work** No difficulty Mild difficulty Moderate difficulty Severe difficulty Extreme difficulty/cannot do Not applicable (e.g. retired or not working prior to injury)	47 (26) 24 (13) 20 (11) 13 (7) 54 (30) 22 (12)	38 (37) 10 (10) 10 (10) 8 (8) 26 (25) 11 (11**)**	0.54

Data are displayed as *n* (%). Data compared with chi-square tests. Missing responses: S2 domain; *Delirium* = 1: S3 domain; *No Delirium* = 1: S4 domain; *Delirium* = 3: S5 domain; *Delirium* = 1: S8 domain; *No Delirium* = 1; S10 domain; *Delirium* = 1, *No Delirium* = 2; S11 domain: *Delirium* = 2; S12 domain; *No Delirium* = 2.

## Discussion

In this prospective multi-site study of severely injured critical care patients, two-thirds of respondents reported having distorted memories or had been told that they were delirious at some point whilst in hospital. There were few characteristic differences between groups except an increased rate of TBI in those who reported delirium. Patients with delirium required longer hospital stays and more injury-related outpatient follow-up post-discharge. At 12 months post-injury in hospital delirium was associated with reduced quality of life and fewer ‘difficulty-free’ days where patients expressed problems with psychological rather than physical functioning. Early identification and modification of delirium risk in critical care trauma patients may improve long-term outcomes, and this requires investigation in larger studies.

Two-thirds of our patients reported experiences of delirium or distorted memories in critical care, which was similar to previous single site trauma studies at 67%^28^ and 61%.^29^ More recently, the incidence appears to have reduced by half, where 36%^30^ or 30%^31^ were reported. In these more recent studies, predictors of delirium included midazolam use^28^ which we did not measure, or mechanical ventilation^30^ and frailty,^31^ neither of which were different across our groups. Only one study, which included patients aged 50 years or older, found an association with age, severe injury and TBI and the development of delirium.^29^

Whilst age and ISS were similar in our two cohorts, rates of TBI were higher in the delirium group. However, the relationship between TBI and delirium is complex, and historically, definitions of delirium emphasise its acute nature not only in terms of onset but also resolution. Delirium is also frequently characterised by its fluctuating presentation. Consequently, there is a focus on the modifiable nature of in hospital delirium, with environmental and pharmacological mitigation promoted, and efforts made to identify patients at higher risk.^32–35^ These features of delirium are less clear cut in cases of TBI, where the longer-term effects of the primary injury and associated brain damage are not easy to distinguish from temporary disorders of consciousness and arousal but are likely to last much longer. There is a risk that in cases of TBI, functional and potentially treatable components of delirium may be under-recognised and go undertreated.^36^ Whilst derangements in cholinergic, serotoninergic and dopaminergic regulatory systems may contribute to any form of delirium, the pathophysiology of delirium in TBI may be distinct and represent a more complex imbalance of neurotransmitters and intra-cortical networks alongside the effects of neuroinflammatory substances, triggered by the primary brain injury and anatomical area of damage.^36^

Only one small prospective observational study focused on a cohort of TBI patients, which found that almost half of patients with mild to moderate head injuries developed delirium within the first 4 days following injury.^37^ Yet many studies of delirium excluded patients with TBI, neurological or neurosurgical disorders entirely,^10,14,17,20,21^ and this may inhibit a better understanding of these complexities. Other studies focussed on critically ill patients in which pre-existing or primary neurologic insult was not explicit,^38^ whilst some excluded patients with suspected pre-existing cognitive impairment.^28,39^ One study included TBI patients but excluded disordered consciousness, which rendered the patient unable to verbalise, which is likely to have included the most severe TBI cases^31^ whilst another excluded those with neurological deficits, which precluded independent living.^18^ There is, therefore, a risk that functional and potentially treatable components of delirium in TBI may be under-recognised and go undertreated.^36^

Notwithstanding TBI, the underlying pathophysiological substrate for long-term cognitive impairment and its relationship to delirium after major trauma also requires further investigation. As different aetiologies lead to a shared, albeit heterogeneous, core syndrome, some researchers have proposed that a common pathogenic pathway underpins delirium: however, evidence is currently lacking for such a single pathway.^40^ Alternatively, there is evidence in mice models that acute systemic inflammation produces both reversible cognitive deficits, resembling delirium, and acute brain injury contributing to longer-term cognitive impairment, but that these events are mechanistically dissociable which could have significant implications for management of cognitive dysfunction during acute illness.^41^ Development of a new paradigm for delirium has been proposed to better explore the heterogeneity of the pathological processes at play, whilst simultaneously focusing on treatment interventions to ameliorate the impact of delirium on a patient's experience, their family's distress, caregiver burden and the course of their care.^42^ However, treating the underlying cause of delirium might not be possible if that cause is elusive, not readily reversible, historical (such as surgery or TBI), or already resolved.^43^

Rates of MODS were high across all patients although more prevalent in the delirium group. MODS developed on the first day following critical care admission for all patient groups,^8^ which is likely earlier than delirium development. Comparison with other delirium studies is limited as many do not disclose MODS, even in critically ill populations. Where reported, incidence of MODS did not differentiate between a delirium and control group,^28^ one study had an increased incidence in their delirium group similar to ours^10^ and one found no difference in MODS between ‘delirium’ and ‘no delirium’ groups.^17^ Whilst it is tempting to associate illness severity (such as MODS) as a predisposing factor for delirium, this may be too simplistic an interpretation,^16^ not least due to heterogeneity in reporting illness onset and severity. We were unable to examine the relationship between time to delirium onset and MODS as this was not measured in our study but may merit further investigation.

Delirium was associated with reduced quality of life at 12 months post-injury, with lower VAS scores, and fewer ‘difficulty free’ days. However, most patients in both groups were able to carry out their usual activities to some extent day to day. Multiple longitudinal studies report incremental improvements in most domains of functioning for trauma populations as time from injury lengthens.^38,44–47^ Results from our study suggest within a single month, daily progress may not be linear. Emotional sequelae are common problems at 12 months,^47^ and this may be more so for patients who experienced in-hospital delirium. Using both measures of outcome, the patients who experienced delirium reported more anxiety, depression and emotional problems at follow-up. Very few trauma studies have examined in-hospital delirium with longer-term psychological outcomes. Where mental health is explored in relation to major physical trauma, the predominant focus is often on manifestations of Post Traumatic Stress Disorder (PTSD) as a direct consequence of injury.^48,49^ This may represent an over-simplification of long-term mental health outcomes, which contributes to an underestimation of the impact of other psychological consequences of trauma-associated critical illness.

There is conflicting evidence regarding the relationship between in-hospital delirium and long-term anxiety and depression, much of it focused upon non-trauma critically ill patient populations. In 382 medical-surgical ICU patients assessed at 12 months post-discharge, longer duration of delirium was associated with higher levels of depression and worse mental health outcomes.^39^ We did not measure duration of delirium, and this warrants further investigation. Whilst patients in our delirium group were more likely to have received some form of support from a healthcare professional trained in psychological therapies, fewer than half did. This is a concerning finding given the higher incidence of emotional and psychological problems reported in this group and may contribute to the reported quality of life reductions.

### Limitations

This study contains some important limitations. Firstly, the low response rate influenced the small size of our overall cohort and therefore the number of the patients in each group. Response rates in longer-term critical care follow-up studies are variable,^50^ and ours may have been affected by the higher proportions of older people enrolled into MODET. Nevertheless, we acknowledge that our 40% response rate may have led to a response bias. Secondly, our method for determining which patients in our cohort experienced delirium relied entirely on retrospective patient self-reporting rather than the use of a specific contemporaneous delirium screening tool. Patient reported outcome measures are currently underutilised in trauma populations and have an important role in capturing and quantifying the patients’ perspective.^51^ In this sense, our study reflects a zeitgeist, which aims to place the patient at the centre of the feedback process. However, there are some significant limitations to patient self-reporting of delirium, not least a poor lay knowledge of delirium among the general populace, which may impact on recognising it as something that has been encountered or experienced. The 12-month timeframe between injury and follow-up may also have increased the potential for recall bias. Finally, our study question focused on distorted memories, confusion and delirium experienced *at any point* during hospitalisation. As such, we cannot claim to have investigated the incidence of delirium occurring exclusively during critical illness. However, delirium is known to have increased prevalence in the critically ill trauma population.^30^ This, coupled with the particularly high incidence of amnesia during critical care reported by patients in the delirium group, suggests a high likelihood that delirium episodes originated in the critical care unit.

## Conclusion

Patient reported delirium was common in a multi-site trauma critical care cohort, associated with lower quality of life and fewer ‘difficulty free’ days at 12 months after injury. TBI incidence was higher in the delirium group and may have been a contributing factor, and this warrants further investigation. Psychological rather than physical recovery problems predominated in our cohort and findings suggest that longer-term psychological support is far from embedded in current models of the UK trauma networks. Finally, further research into delirium subtypes may add to the understanding of different aetiologies, provide opportunities for effective targeted interventions or preventative measures, and improve outcomes for patients, their families and caregivers.
